# Generation of metastatic melanoma specific antibodies by affinity purification

**DOI:** 10.1038/srep37253

**Published:** 2016-11-17

**Authors:** Birgit Schütz, Anita Koppensteiner, David Schörghofer, Katharina Kinslechner, Gerald Timelthaler, Robert Eferl, Markus Hengstschläger, Albert Missbichler, Harald Hundsberger, Mario Mikula

**Affiliations:** 1 Institute of Medical Genetics, Medical University of Vienna, Vienna, Austria; 2 Medical and Pharmaceutical Biotechnology, IMC University of Applied Sciences, Krems, Austria; 3 Institute of Cancer Research, Medical University of Vienna and Comprehensive Cancer Center (CCC), Vienna, Austria; 4 Sciotec Diagnostics Technologies GmbH, Tulln, Austria

## Abstract

Melanoma is the most aggressive type of skin cancer and one of the most frequent tumours in young adults. Identification of primary tumours prone to develop metastasis is of paramount importance for further patient stratification. However, till today, no markers exist that are routinely used to predict melanoma progression. To ameliorate this problem, we generated antiserum directed against metastatic melanoma tissue lysate and applied a novel approach to purify the obtained serum via consecutive affinity chromatography steps. The established antibody, termed MHA-3, showed high reactivity against metastatic melanoma cell lines both *in vitro* and *in vivo*. We also tested MHA-3 on 227 melanoma patient samples and compared staining with the melanoma marker S100b. Importantly, MHA-3 was able to differentiate between metastatic and non-metastatic melanoma samples. By proteome analysis we identified 18 distinct antigens bound by MHA-3. Combined expression profiling of all identified proteins revealed a significant survival difference in melanoma patients. In conclusion, we developed a polyclonal antibody, which is able to detect metastatic melanoma on paraffin embedded sections. Hence, we propose that this antibody will represent a valuable additional tool for precise melanoma diagnosis.

Melanoma is the most aggressive type of skin cancer with alarmingly increasing incidences in the Caucasian population^1^,^2^. Indeed, it represents the most rapidly growing cancer incidence rate in men and the second fastest in women after lung cancer^3^. At early stages, melanoma is curable by surgical intervention alone and, accordingly, 5-year survival rates at these stages peak up to 97%^4^. However, melanoma can metastasize to other parts of the body, like lymph nodes, lung and brain. Under these circumstances, 5-year survival rates drop drastically to as low as 10%^4^. Indeed, melanoma metastases are the major cause of death and, thus, early detection and diagnosis accompanied by careful selection of patients for adjuvant treatment are crucial factors in melanoma therapy. However, identification of patients with high risk for metastasis proves difficult.

Immunohistological staining is a frequently used tool for cancer diagnosis. The most commonly used immunohistological biomarkers for detection of melanoma are S100b, HMB-45, Melan-A and tyrosinase^5^,^6^,^7^,^8^, with S100b being the most sensitive one^9^,^10^. Nevertheless, due to its lack of specificity, S100b is predominantly used in combination with other biomarkers.

To our knowledge, so far no antibody exists which can predict the metastatic potential of melanoma. Therefore, in this study, we aimed to generate an antibody able to detect melanoma prone to metastasis. We hypothesized that isogenic human melanoma cell lines grown in mice represent human melanoma characteristics and that immunization of rabbits with this tumour lysate would raise antibodies directed against metastatic melanoma epitopes. To enhance serum specificity we applied a sophisticated affinity purification strategy to yield an antibody termed MHA-3.

## Results

### Generation of polyclonal serum and affinity purification

**Figure 1:**
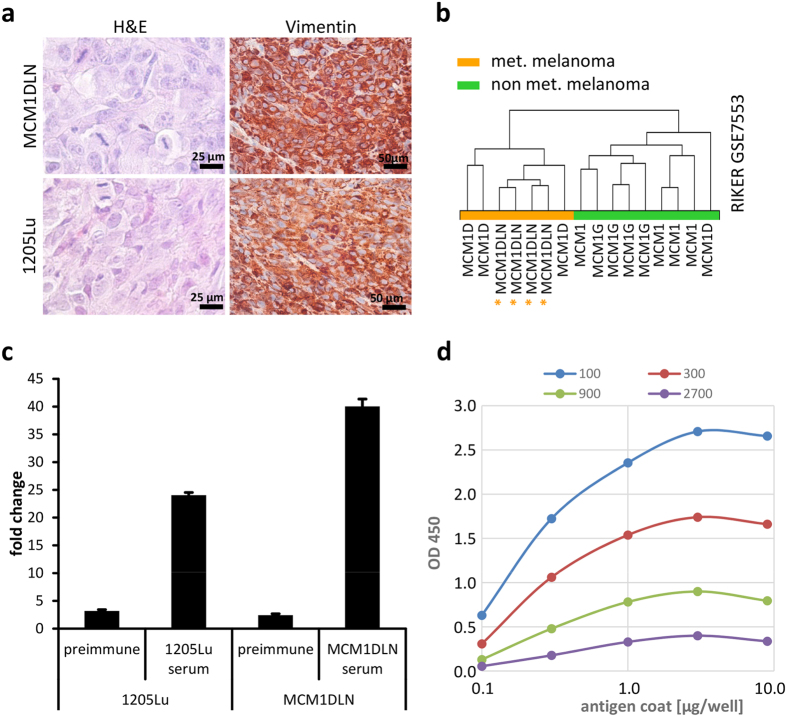
Immunization with metastatic melanoma antigen. (**a**) Hematoxylin eosin stain (H&E) and human specific vimentin staining (red) of MCM1DLN and 1205Lu xenotransplanted tumours. (**b**) Unsupervised clustering of MCM1DLN tumours according to expression signature derived from 40 metastatic melanoma lesions versus 16 primary melanomas (fold change >2, p < 0.05) performed by Transcriptome Analysis Console software with no filtering. (**c**) ELISA coated with 1 μg of tumour lysate (1205Lu and MCM1DLN) incubated with respective serum derived either prior (preimmune) or after immunization. Fold changes relative to negative control are shown. (**d**) Dose response curve showing the OD450 values for antigen coating concentration versus antibody dilution factor.

For generation of metastatic melanoma antigens we used established and described metastatic melanoma cells MCM1DLN and 1205Lu^11^,^12^. Cells were intradermally transplanted into immune-compromised mice and after 4 weeks tumours were resected. Hematoxylin and eosin staining of tumours revealed presence of a heterogeneous cell population with multiple mitotic figures, indicating an aggressive phenotype (Fig. 1a). Further, staining of tumours for human specific vimentin revealed that the majority of cells are of human origin while few unstained cells are mouse stroma-derived. Generally, MCM1DLN tumours displayed characteristics of human primary melanomas which were able to form metastases. This could be shown by using a metastasis specific gene signature derived from GSE7553, which assigned MCM1DLN tumours to the metastatic melanoma group by unsupervised clustering, while MCM1 tumours were assigned to the non metastatic melanoma group (Fig. 1b). Next, whole tumours were lysed and used for repeated immunization of rabbits. ELISA assay was used to assess successful antibody generation (Fig. 1c). Serum collected from immunized rabbits yielded a 5 times higher titer in the ELISA assay than serum taken before immunization (pre-immune) for the 1205Lu cell line and a more than 20 times higher titer for MCM1DLN. To confirm signal specificity we performed dose response testing (Fig. 1d). For all dilutions tested signal intensity was proportional to the antigen concentration used, except for the highest antigen dose which showed effects of saturation.

**Figure 2:**
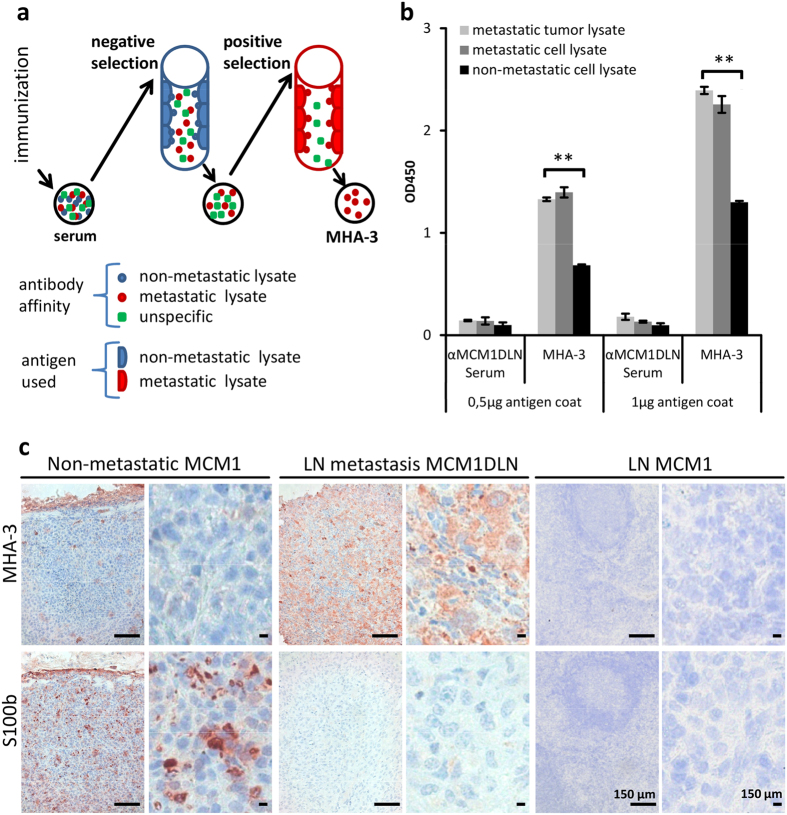
Affinity purification process. (**a**) Schematic representation of applied method. Serum after immunization contains antibodies against metastatic (red) and non-metastatic (blue) melanoma as well as non-specific antigens (green). Serum is first negatively selected by removing antibodies that bind to antigens from non-metastatic melanoma cells. Next, non-binding antibodies are positive selected by binding to metastatic melanoma antigens. The eluate is termed MHA-3. (**b**) ELISA coated with metastatic tumour and cell lysate and non-metastatic cell lysate at two different concentrations (0.5 and 1 μg/well). MCM1DLN serum and MHA-3 are used as primary antibodies. Experiments were done in triplicates and one-way Anova test was performed (**p < 0.01). Error bars represent standard error of mean. (**c**) Representative immunohistochemical images (from five tumour samples each) of MCM1 tumour (left), lymph node (LN) metastasis of MCM1DLN tumour (middle) and LN of MCM1 tumours (right) stained with S100b and MHA-3.

A schematic description of our novel approach to process antiserum by the use of two affinity columns is shown in Fig. 2a. First, we performed negative selection to deplete for antibodies able to bind non-metastatic lysates (WM793b or MCM1). This was done by covalent binding of the cell lysate to the column and subsequent incubation with antiserum. Antibodies not binding to the column were collected and used for the second chromatography step. There, antibodies able to bind metastatic tumour lysates (either 1205Lu or MCM1DLN derived) were positively selected. This was achieved by eluting antibodies under low pH conditions. ELISA was used to determine the eluate fraction with the highest affinity to the antigen (Supplementary Fig. S1a). All experiments were performed with two sets of isogenic cell lines and two different immune sera. Since MCM1DLN-derived tumours generated the strongest immune response, the affinity derived antibody thereof was used for further analysis and termed MHA-3.

### Characterisation of MHA-3

To compare MHA-3 to the originally obtained serum in terms of antigen binding, we performed ELISA with two different concentrations of antigen coating. We used lysates from metastatic tumours as well as metastatic and non-metastatic cell lysates. To ensure that both primary antibodies were used in the same immunoglobulin concentration, a titration ELISA was performed (Supplementary Fig. S1b). We could show that MHA-3 binds the metastatic lysates significantly better than the non-metastatic lysates (p < 0.01) regardless of the antigen coating concentration (Fig. 2b).

To further investigate the ability of our generated antibody to detect metastatic melanoma cells we performed immunostainings with MHA-3 and S100b (Fig. 2c). We detected positive staining of lymph node metastases from mice injected with metastatic MCM1DLN cells. Primary tumours from mice injected with non-metastatic MCM1G cells were only weakly positive for MHA-3. Contrary, S100b staining was highly positive in the non-metastatic tissue samples, whereas it was nearly absent in the metastatic tissue samples. We used lymph nodes from mice, previously injected with MCM1 cells as staining controls. Neither MHA-3 nor S100b showed a postive signal in these sections.

### Association of clinicopathological parameters with MHA-3 expression

To validate our findings, we assessed MHA-3 protein expression in melanoma and lymph node metastasis samples derived from human patients. Clinical and pathological characteristics of melanoma patients are summarized in Table 1. A total of 261 biopsies were subjected to immunohistochemical staining for MHA-3 and consecutive sections from the same patients were simultaneously stained with S100b.

**Figure 3:**
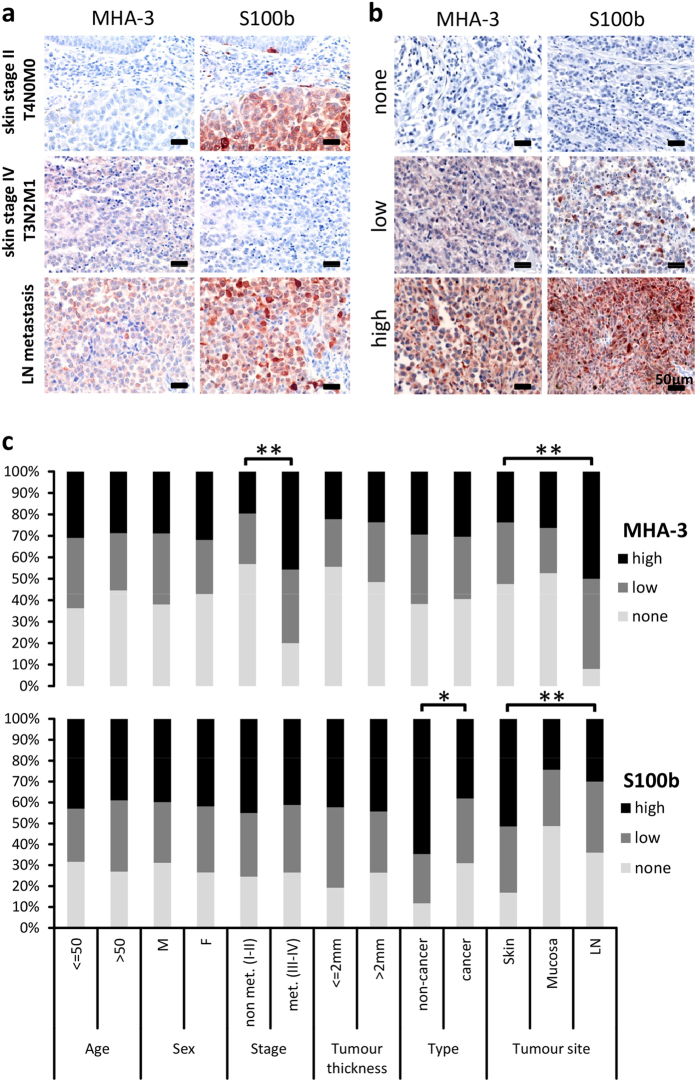
Association of MHA-3 staining with human melanoma. (**a**) Staining distribution of MHA-3 and S100b in low and high grade melanoma and lymph node biopsies. (**b**) Representative examples for scoring of MHA-3 and S100b staining are shown. (**c**) The overall scoring distribution for MHA-3 and S100b staining intensity in different clinicopathological groups. Unstained samples are shown in light grey, low staining in grey and high staining intensity in black. Clinicopathological groups along with age and sex of the patients are plotted on the x-axis. P-values are shown in the graph marked *for p < 0.01 and **for p < 0.0001.

Figure 3a shows staining characteristics of MHA-3 and S100b at different stages of melanoma development. Furthermore, the samples were scored according to their staining intensity. Representative cores for different staining intensities are shown in Fig. 3b. On the basis of this classification system we evaluated associations between MHA-3 or S100b and the presence of clinicopathological parameters, including pathological type, clinical stage and lymph node metastasis (Fig. 3c). There, MHA-3 was able to discriminate non-metastatic tumour stage from metastatic tumour stage (p < 0.001) as well as tumours residing in the skin from tumours residing in the lymph node (p < 0.001), but MHA-3 was not able to distinguish cancer from non-cancer. S100b showed significant weaker staining in lymph nodes compared to primary tumours (p < 0.01).

### Sensitivity and specificity determination

**Figure 4:**
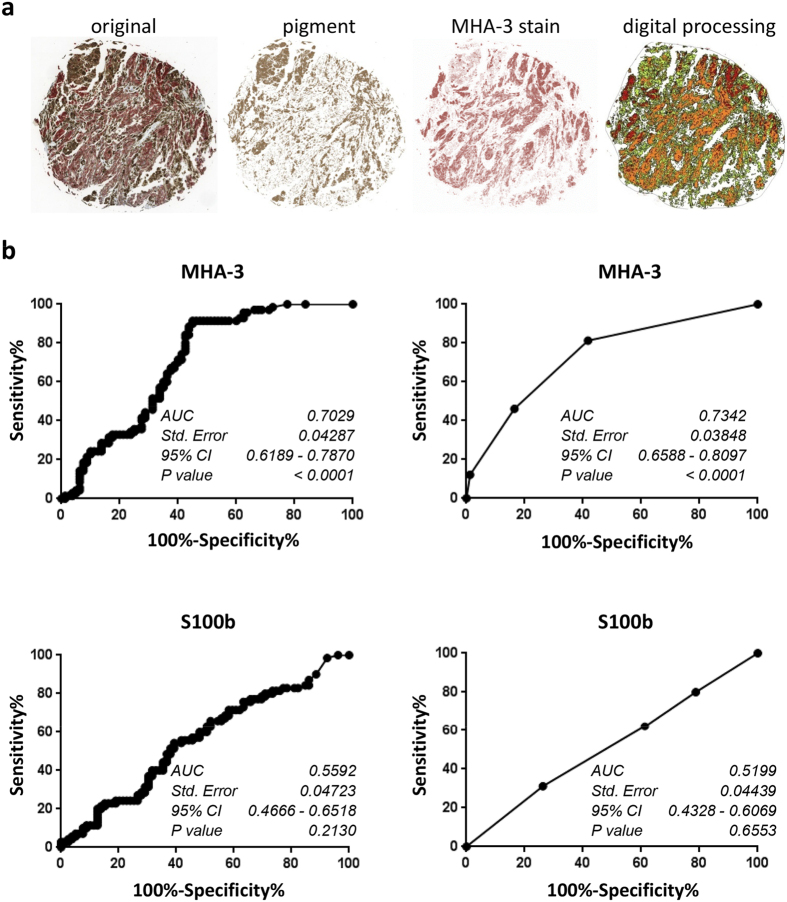
Detection of melanoma metastasis by MHA-3. (**a**) Example of digital processing for staining quantification. The cores of the melanoma arrays (n = 236) were scanned and subjected to colour deconvolution, which removed brown pigment stain and allowed software-assisted quantification of positive area (shown in red and orange colour). (**b**) ROC curves were calculated based non-metastatic melanoma vs. metastatic melanoma staining intensities for MHA-3 and S100b. ROC curves on the left were derived from digital images, whereas the curves on the right were derived from two independent observers.

Since the original scores were observer-derived, we chose to additionally evaluate our samples also in an observer-independent way. Therefore, we generated high resolution images and performed colour deconvolution on each biopsy core (Fig. 4a). Next, we calculated the percentage of positive staining area compared to the whole tissue area. Subsequently, samples were grouped into cohorts consisting of non-metastasizing primary melanoma and of metastasizing primary melanoma as well as lymph node metastases. Using the quantification data derived from image analysis, we generated ROC curves for MHA-3 and S100b once by digital image processing and once by the aforementioned staining scores (Fig. 4b,c).

Next, we calculated the area under the curve (AUC) for both the observer-dependent and the observer-independent analysis. Both evaluation methods gave very similar results. Statistical analysis revealed an AUC for MHA-3 of approximately 0.7 with a p-value below 0.001 in both quantifications. Furthermore, we determined the optimal cut-off for MHA-3 staining evaluation at 3.9, with a sensitivity of 91% and a specificity of 56%. For S100b we determined an AUC of only 0.52 to 0.56 which resulted in a p-value of 0.213 for observer-dependent and a p-value of 0.655 for observer-independent analysis.

### Antigen identification

**Figure 5:**
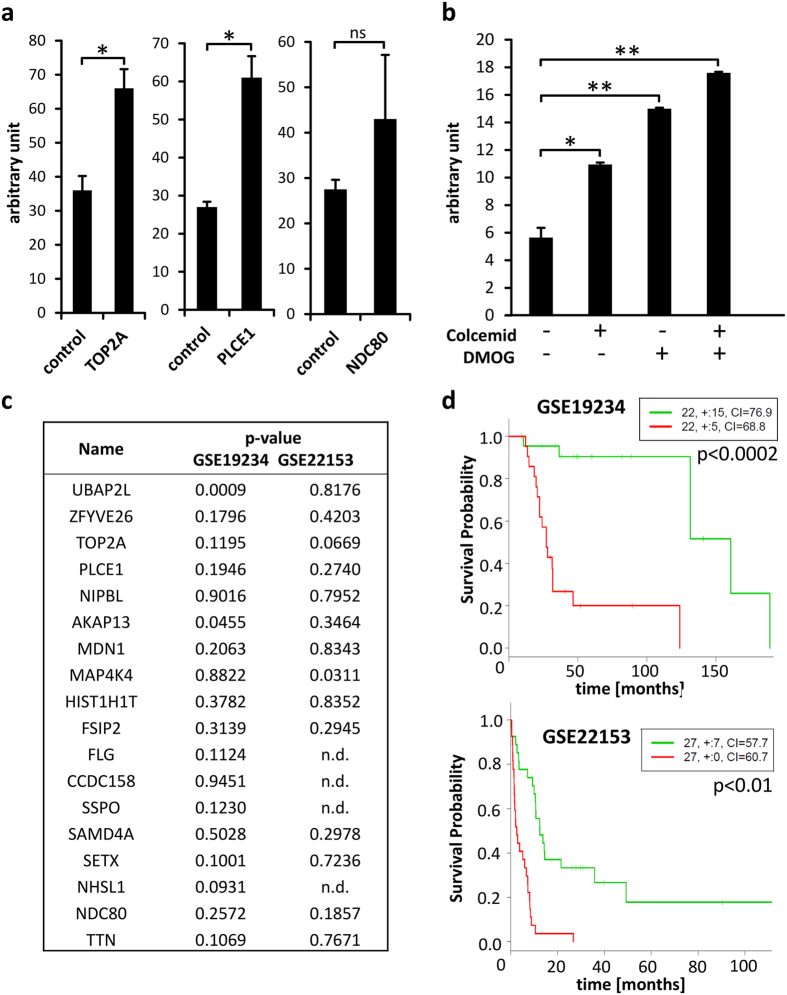
MHA-3 target validation. (**a**) ELISA plate was coated with recombinant TOP2A, PLCE1 and NDC80, detected by MHA-3 and compared to BSA control. (**b**) MCM1 cells were treated with either colcemid or DMOG or both and subjected to ELISA measurement by MHA-3 incubation. All experiments were performed in triplicates and unpaired t test was used to compare the results (*p < 0.05 and **p < 0.01). Error bars represent standard error of mean. (**c**) p-values are given for Kaplan Meier analysis in two independent patient cohorts (GSE19234 and GSE22153) for each of the 18 identified proteins. (**d**) Kaplan Meier curves from SurvExpress analysis showing the p-value after log-rank testing for all identified target proteins combined. Concordance Index was 83.68 and 71.58 for GSE19234 and GSE22153, respectively. Red and green curves denote high- and low-risk groups, respectively.

Immunization with whole tumour lysate led to the generation of antibodies directed against multiple targets. To identify antigens bound by MHA-3, we performed immunoprecipitation followed by mass spectrometry. Using a minimum recognition rate of three peptides per protein led to the identification of 18 target proteins (Table 2). Of these we used recombinant DNA topoisomerase 2-alpha (TOP2A), kinetochore protein NDC80 homolog (NDC80) and 1-phosphatidylinositol 4,5-bisphosphate phosphodiesterase epsilon-1 (PLEC1) to validate MHA-3 binding and we observed increased binding efficiency compared to normalized BSA control samples (Fig. 5a). Interestingly, TOP2A, NDC80 and A-kinase anchor protein 13 (AKAP13) belong to the hallmark geneset termed MITOTIC_SPINDLE. Additionally, AKAP13 and Filaggrin (FLG) expression was demonstrated to depend on hypoxia-inducible factor (HIF) signalling^13^,^14^. Therefore, we used colcemid, a microtubule depolymerising drug, as well as the HIF stabilizing agent dimethyloxalylglycine (DMOG) to treat melanoma cells and to harvest protein lysates. We used 2 ng/ml of colcemid since it did not alter tubulin levels, but it increased cell migratory capabilities (Supplementary Fig. 2). Inhibition of Prolyl Hydroxylase Domain (PHD) enzymes by DMOG incubation led to a strong increase in HIF1A amounts (Supplementary Fig. 2). ELISA analysis revealed MHA-3 binding to increase upon individual colcemid or DMOG treatment and maximum binding was observed when cells were treated with both colcemid and DMOG (Fig. 5b). Finally, we used available expression data from two independent studies to analyse patient survival according to expression of our identified proteins^15^,^16^. Of the 18 identified targets, none showed significant prediction of patient survival in both cohorts (Fig. 5c). However, when we used expression of all 18 proteins together, we could estimate survival with p < 0.0002 in GSE19234 and p < 0.01 in GSE22153 (Fig. 5d).

## Discussion

Here, we used a novel approach for the generation of metastatic melanoma specific antibodies. We immunized rabbits with lysed, metastatic melanoma tissue and depleted the generated serum of antibodies reactive against isogenic, non-metastatic tumour cells. This procedure yielded the MHA-3 serum antibody, which was able to detect human metastatic melanoma within our experimental settings. Furthermore, we identified antigens bound by MHA-3 and we showed that HIF inducers as well as tubulin destabilizing agents have the potential to induce antigenicity in non-metastatic melanoma. Importantly, our identified antigens have the potential to predict patient survival time.

To define whether melanoma and especially thin melanoma ( = <1 mm) will undergo progression is still a major research question. Clinical data collection and histopathologic analysis has contributed to estimate the survival rate of patients^4^. Still, currently there is no marker in clinical use, which shows high prognostic accuracy. Routine immunohistochemical staining for diagnosis of metastatic melanoma primarily relies on melanoma differentiation markers^6^. While S100b is the most sensitive of those markers, others, like HMB45, Melan-A or tyrosinase show higher specificity^7^,^8^. However, HMB45 and tyrosinase have been reported to have decreased sensitivity in metastatic melanoma, making it difficult to use them as single diagnostic markers^7^,^17^. To further distinguish benign nevi from melanoma the proliferation marker Ki-67 is commonly used^18^.

The novel antibody MHA-3 was generated by sequential affinity-purification, which turned out to be a highly effective method of polyclonal serum modification. The purification process resulted in a significant reduction of unspecific binding and enrichment in binding affinity to metastatic melanoma antigens, which could be verified in ELISA measurements as well as in immunohistochemical stainings. Melanoma patient sample analysis revealed a strong association of MHA-3 with the tumour site and was able to identify lymph node metastasis, which was not possible with S100b. Furthermore, MHA-3 staining was associated with tumour stage, recognizing predominantly stages III and IV. Additionally, we observed higher, albeit not significant, MHA-3 staining in thicker melanoma tumours. It is possible and remains to be determined, whether MHA-3 staining is able to detect single cells prone to develop metastasis at an early tumour stage.

Most of the new biomarkers that have been developed recently are based on mRNA expression profiles from melanoma cell lines or metastatic tumour samples. Contrary, here we used *in-vivo* grown human melanoma tissue, which closely mimics the full complexity of human patient material. Additionally, our approach is proteome-based and, hence, not limited to the detection of changes on mRNA level. Potentially, our diagnostic serum is therefore superior to classical antibody biomarkers since it originates from a more holistic approach.

In order to gain insights on the metastatic phenotype, we further investigated antigens bound by MHA-3. We identified an overlap with the geneset HALLMARK_MITOTIC_SPINDLE based on the presence of TOP2A, NDC80 and AKAP13. Importantly, TOP2A is also part of the DNA repair processes and has been described to be associated with melanoma metastasis^19^,^20^,^21^,^22^,^23^,^24^. This is of interest, since we already observed a high frequency of mitotic figures in tumours used for immunization, indicating either active cytokinesis or increased DNA repair. Furthermore the genes TOP2A, NDC80 and mitogen-activated protein kinase kinase kinase kinase (MAP4K4) belong to the geneset WINNEPENNINCKX_MELANOMA_METASTASIS_UP. This geneset contains up-regulated genes in melanoma patients with a reported distant metastasis within 4 years^19^. NDC80, PLCE1 and AKAP13 have so far not been studied in melanoma. However, in several other cancers their expression was linked to a worsened prognosis^25^,^26^,^27^,^28^,^29^,^30^. These findings are also complemented by the use of the drug colcemid, which interferes with mitotic spindle formation. Low doses of colcemid have been shown to moderately effect the mitotic index and low doses could also enable the transformation of fibroblasts^31^,^32^. Furthermore we enriched for HIF1A protein prior to testing for MHA-3 reactivity, since HIFs have been shown to be instrumental for driving melanoma metastasis^33^. In conclusion our results show that the metastatic melanoma phenotype is characterized by expression of proteins involved in mitosis and HIF signalling and that simultaneous expression profiling of all 18 proteins is able to predict patient survival.

The importance of accurate melanoma metastasis risk prediction is underscored by the fact that in a population with a very high incidence of melanoma and a long history of melanoma education, more people die from thin melanoma than from thick melanomas^34^. Therefore it is of uttermost importance to differentiate curable melanomas from those that will recur with metastasis^35^. We hope that with the development of our novel antiserum we can contribute to the identification of patients with high mortality risks, which require further adjuvant therapy.

## Methods

### Melanoma cell culture

WM793b and 1205Lu cells were obtained from American type Culture Collection (ATCC, Manassas, VA). MCM1 and MCM1DLN were patient derived as previously described^12^. Cells were grown in 2% MIM (80% MCDB, 20% Leibovitz’s L-15, 2% FBS, 5 μg/ml Insulin, 5 ng/ml EGF, 1.68 mM CaCl_2_) supplemented with 50 mg/L streptomycin sulphate and 30 mg/L penicillin until 90% confluent. Cells were dissociated in 0.25% trypsin/EDTA and counted with a CASY cell counter. Cells and tumours were lysed in lysis buffer (40 mM Hepes, 120 mM NaCl, 1 mM EDTA, 10 mM 2-Glycerophosphate, 50 mM NaF, 0.5 mM Na_3_VO_4_ and 1% NP-40) using a Precellys^®^ homogenizer. Protein concentrations were determined by Bradford analysis.

### Colcemid/DMOG treatment

MCM1 cells were cultured 70% confluent in 2%MIM. Cells were treated with 0.2 ng/ml colcemid or 1 mM DMOG or with a combination of both. After 72 h cells were lysed for ELISA with MHA-3 as described above.

### Generation of tumours in mice and immunization of rabbits

For transplantation 3 million cells were diluted in 100 μl PBS and intra dermally injected into eight-week-old CB.17 SCID/SCID female mice (Charles River, L’Arbresle, France). Metastatic cell lines (MCM1DLN and 1205Lu) grew tumours after approximately 4 weeks and were collected for immunization. Non-metastatic cell lines MCM1G formed tumours after 8 weeks. Adult female New Zealand White rabbits were used for raising antisera directed against the tumour lysates of the metastatic cell lines. Rabbits were immunized by injecting a mixture of 250 μg antigen and adjuvant in subcutaneous sites and booster immunizations were given in intervals of two weeks. After test bleeds confirmed the immune response via ELISA, serum was collected and stored at −20 °C. All animal procedures were approved by the “Animal Care and Use Committee” of the Medical University of Vienna. All methods were carried out in accordance with the approved guidelines of the Animal Care Committee.

### ELISA

Cell and tumour lysates or recombinant proteins were coated onto Nunc^®^ Maxisorp plates at a concentration of 0.5 μg per well and incubated over night at 4 °C. After 1 hour of blocking in 5% BSA the crude serum or isolated antibody fractions were added in a 1/50 dilution in 1.5% BSA. Plates were again incubated over night at 4 °C and HRP-labelled secondary antibody was added for 1 hour at RT. The reaction was visualized by TMB substrate and stopped using 2 M H_2_SO_4_. Absorbance was determined at 450 nm and 630 nm served as reference. For measuring of immunoglobulin concentration the serum was coated directly to the plate and detected with a second-step antibody. All other steps are the same as for the detection ELISA.

### Affinity chromatography

The rabbit serum containing the desired antibodies was purified using an ÄKTAFPLC (GE Healthcare). Therefore each cell line or tumour derived lysate was diluted in coupling buffer containing 0.2 M NaHCO_3_ and 0.5 M NaCl, pH 8.3 to a final concentration of 1 mg/ml and packed into a 1 mL HiTrap^TM^ NHS-activated HP column. Next, serum was diluted 1/10 and applied to the respective column via a superloop. PBS was used as binding buffer. Antibodies that did not bind remained in the flow through and were collected (called negative selection). Antibodies from the flow through were bound to an appropriate second column and eluted using 0.2 M Glycine, pH 2 and collected in 1 mL fractions (called positive selection). Antibody solution was neutralized by 1 M Tris Buffer, pH 9 to stabilize the proteins.

### Immunohistochemical analysis and colour deconvolution

Tumours were fixed, dehydrated and embedded in paraffin. Paraffin was removed using Xylol, Isopropanol and an alcohol gradient in descending order. For antigen retrieval the slides were incubated in modified citrate buffer, pH 6, at 120 °C. Slides were incubated in 1% H_2_O_2_ and permeabilized with 0.1% Tween 20. Primary antibodies, S100b (1/20000; Dako) and MHA-3 (1/50), were incubated overnight at 4 °C. After washing with PBS, a biotinylated secondary antibody was added for 45 min and slides were incubated with Streptavidin-HRP complexes conjugated with peroxidase. To visualize positive staining, sections were incubated with Aminoethyl carbazole for 20 min and hemtaoxylin was used for counterstaining the nuclei. Immunostaining of the anti-S100b antibody and MHA-3 was scored by using the following arbitrary scale: no staining (0), low staining, and high staining. Evaluation of tissue sections was performed by two independent researchers, who were blinded regarding patient details.

For digital analysis, melanoma tissue arrays were scanned in high resolution (3DHistech^®^ Pannoramic Slidescanner) and every core was subjected to colour deconvolution. Vectors for pigmentation and Aminoethyl carbazole were defined in ImageJ and images solely representing antibody staining were generated. These pictures were then analyzed with Tissue Studio Software^®^ (Definiens) and the percentage of stained area compared to total area were defined for each core.

### Patient cohort and pathology

All methods involving human patient samples were approved by the institutional ethics review board from the Medical University of Vienna and carried out in accordance with the appropriate guidelines. All patient samples were obtained as anonymized tissue microarrays from US Biomax. Samples were formalin-fixed less than 10 min after surgery, paraffin embedded and assembled as cores with a diameter of 1.5 mm. Tissue sections were quality controlled and contained normal skin tissue and melanoma tissue, representing different stages of disease progression.

### Immunoprecipitation and MS analysis

50 μg protein lysate was pre-cleared with protein A dynabeads^®^ for 30 min. Then the protein was immunoprecipitated with 1 μg of MHA-3 antibody overnight at 4 °C. Immune complexes were bound via incubation with protein A beads for 3 h at 4 °C. Tubes containing the solution were placed on a magnet and unbound protein was discarded and the pellet was washed 5x with cell lysis buffer. The beads were eluted with 0.2 M glycine, pH 2 by incubating the sample for 10 min with agitation before gentle centrifugation. The solution was immediately neutralized with Tris, pH 8. Samples were loaded onto a 10% polyacrylamide gel and following electrophoresis stained with coomassie brilliant blue. Five lanes were cut out and proteins were identified by MS/MS analysis. Scaffold (version Scaffold_4.4.7, Proteome Software Inc., Portland, OR) was used to validate MS/MS based peptide and protein identifications. Peptide identifications were accepted if they could be established at greater than 80.0% probability by the Scaffold Local FDR algorithm. Protein identifications were accepted if they could be established at greater than 95.0% probability and contained at least 3 identified peptides. Protein probabilities were assigned by the Protein Prophet algorithm^36^. Proteins that contained similar peptides and could not be differentiated based on MS/MS analysis alone were grouped to satisfy the principles of parsimony.

### Statistical analysis

All experiments were performed in triplicates and data were averaged and are presented as mean + SEM. Two tailed p-values were calculated by performing unpaired (independent) t-test in SPSS, while one-way Anova was used for comparing three values. P-values ≤ 0.05 were considered to be statistically significant (*) and p < 0.001 was considered as highly significant (**). For analysis of immunohistological staining results the internet tool VassarStats (http://vassarstats.net/index.html) was used. Fisher’s exact probability test for a two-rows by three-columns contingency table was applied to determine association of clinicopathological parameters with MHA-3 and S100b expression defined as none, low or high. Kaplan Meier analysis was performed with the SurvExpress Tool^37^ using datasets provided by Bogunovic *et al.* (44 samples, GSE19234) and Jönsson *et al.* (57 samples, GSE22153). For the analysis, samples were split by the median according to the prognostic index resulting in low- and high-risk groups. Overall performance of the proteins as biomarkers was assessed by the concordance index. Statistical significance was calculated using the log-rank test. ROC analysis and corresponding statistics were calculated using GraphPad Prism Version 6.

## Additional Information

**How to cite this article**: Schütz, B. *et al.* Generation of metastatic melanoma specific antibodies by affinity purification. *Sci. Rep.* **6**, 37253; doi: 10.1038/srep37253 (2016).

**Publisher’s note:** Springer Nature remains neutral with regard to jurisdictional claims in published maps and institutional affiliations.

## Supplementary Material

Supplementary Information

## Figures and Tables

**Table 1:** Clinical and pathological characteristics of patients used for MHA-3 and S100b staining.

Patient Data	Levels	total n
Age (years)	< = 50	116
	>50	145
Sex	M	142
	F	119
Clinical stage	I-II	102
	III-IV	35
Tumour thickness	< = 2 mm	26
	> 2 mm	107
Type	non-cancer	34
	cancer	227
Tumour site	skin	101
	mucosa	76
	LN	50

**Table 2:** Summary of MHA-3 bound antigens identified by MS analysis.

Accession #	Name	Abbreviation	Coverage
O14777	Kinetochore protein NDC80 homolog	NDC80	10.30%
P22492	Histone H1T	HIST1H1T	15.00%
Q5CZC0	Fibrous sheath-interacting protein 2	FSIP2	0.88%
O95819	Mitogen-activated protein kinase kinase kinase kinase	MAP4K4	2.91%
A2VEC9	SCO-spondin	SSPO	1.22%
P11388	DNA topoisomerase 2-alpha	TOP2A	2.42%
P20930	Filaggrin	FLG	1.60%
Q8WZ42	Titin	TTN	0.24%
Q14157	Ubiquitin-associated protein 2-like	UBAP2L	6.26%
Q9UPU9	Protein Smaug homolog 1	SAMD4A	8.91%
Q7Z333	Probable helicase senataxin	SETX	2.05%
Q5SYE7	NHS-like protein 1	NHSL1	3.54%
Q9P212	1-phosphatidylinositol 4,5-bisphosphate phosphodiesterase epsilon-1	PLCE1	1.91%
Q9NU22	Midasin	MDN1	1.13%
Q12802	A-kinase anchor protein 13	AKAP13	1.78%
Q5M9N0	Coiled-coil domain-containing protein 158	CCDC158	4.49%
Q6KC79	Nipped-B-like protein	NIPBL	1.57%
Q68DK2	Zinc finger FYVE domain-containing protein 26	ZFYVE26	2.17%
