# Supplementary material for: Generation of metastatic melanoma specific antibodies by affinity purification
Source: Sci Rep. 2016 Nov 17;6:37253. doi: 10.1038/srep37253 (PMC5112778; doi:10.1038/srep37253)
Supplement: Supplementary Information [file srep37253-s1.pdf]

Generation of metastatic melanoma specific antibodies by affinity purification

Birgit Schütz[1], Anita Koppensteiner[2], David Schörghofer[1], Katharina Kinslechner[1], Gerald Timelthaler[3], Robert Eferl[3], Markus Hengstschläger[1], Albert Missbichler[4], Harald Hundsberger[2], Mario Mikula[1*]

[1] Institute of Medical Genetics, Medical University of Vienna, Vienna, Austria
[2] Medical and Pharmaceutical Biotechnology, University of Applied Sciences, Krems, Austria
[3] Institute of Cancer Research, Medical University of Vienna, Vienna, Austria
[4] Fianostics Ges.m.b.H. Wr. Neustadt, former address: Sciotec Diagnostic Technologies GmbH, Tulln, Austria

*Correspondence to: Mario Mikula, Institute of Medical Genetics, Medical University of Vienna, Währinger Strasse 10, 1090 Vienna. Tel. +43 1 40160 56540; Fax. +43 1 40160 956531; e-mail: mario.mikula@meduniwien.ac.at

Supplementary Methods:

Western blot

MCM1 and WM793b cells were cultured until 70% confluent in 2%MIM. Cells were treated with colcemid (200ng, 20ng, 2ng, 0.2ng and 20pg) or 1mM DMOG or with a combination of both. After 72h cells were either lysed for western blot analysis using antibodies against HIF1A (Santa Cruz) and α-tubulin. To ensure equal loading of the western blot membrane, whole protein imaging was done using the stain-free technology of the imager (BioRad). Membranes were incubated with HRP-linked heavy and light chain secondary antibodies (Thermo Scientific). ECL (Advansta) was used for detection.

Migration assay

For assessment of cell migration cells were seeded onto transwell chambers (Sigma-Aldrich) with 10% FCS used as attractant. After 8 hours of culturing migrating cells were fixed in ethanol, stained with crystal violet and counted.

Supplementary Figure S1

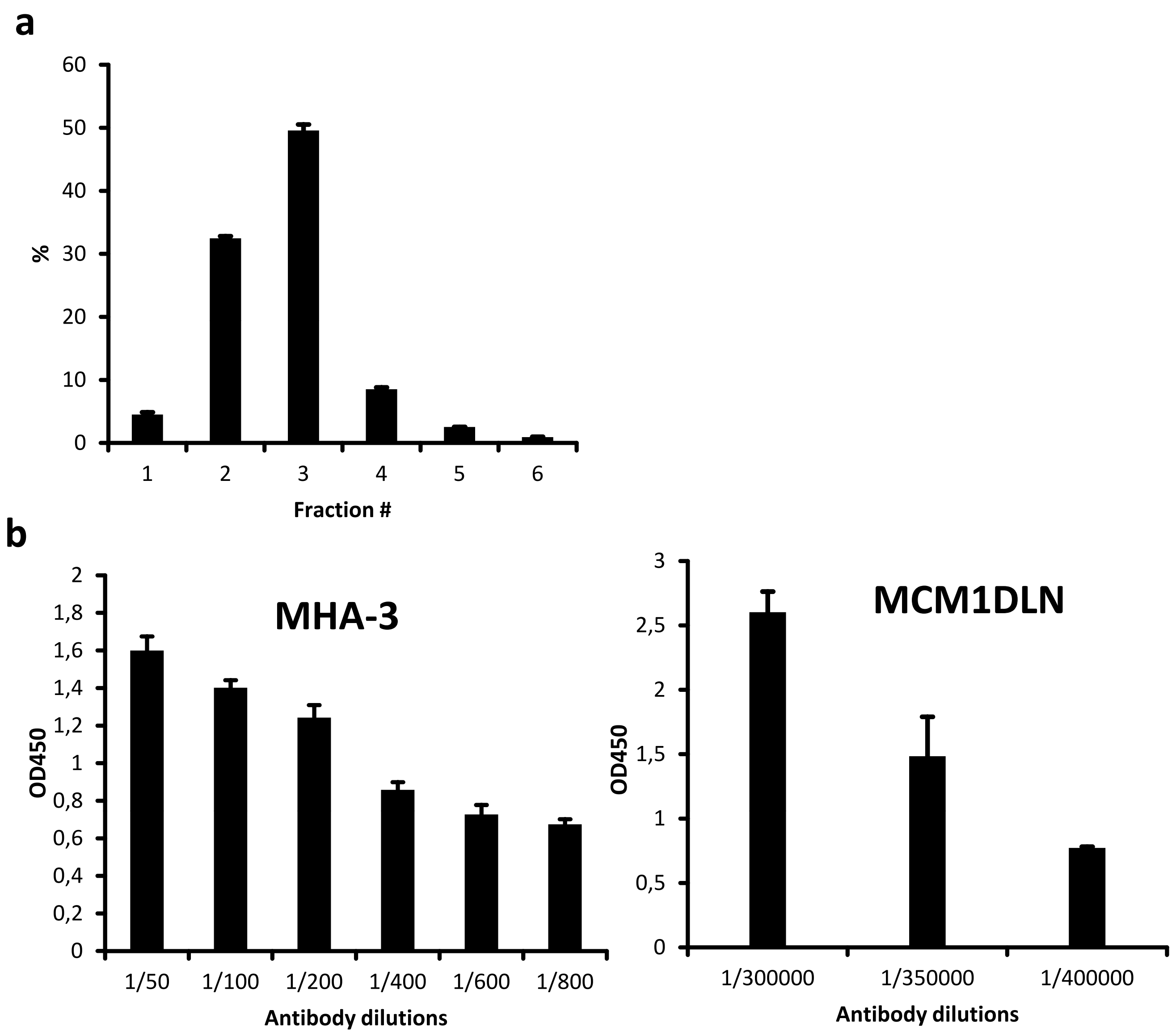

Legend to Supplementary Figure S1:
a) Affinity chromatography results in 6 fractions. Each sample is tested to see the amount of IgG identifying which fraction contains the desired antibody. In all experiments that were performed fraction 3 showed the highest OD450. b) Antibody dilutions were tested for MHA-3 and MCM1DLN antiserum titer and OD450 values were compared. Those dilutions that give a similar OD value were then used for further analysis (1/100 for MHA-3 and 1/350000 for MCM1DLN).

Supplementary Figure S2

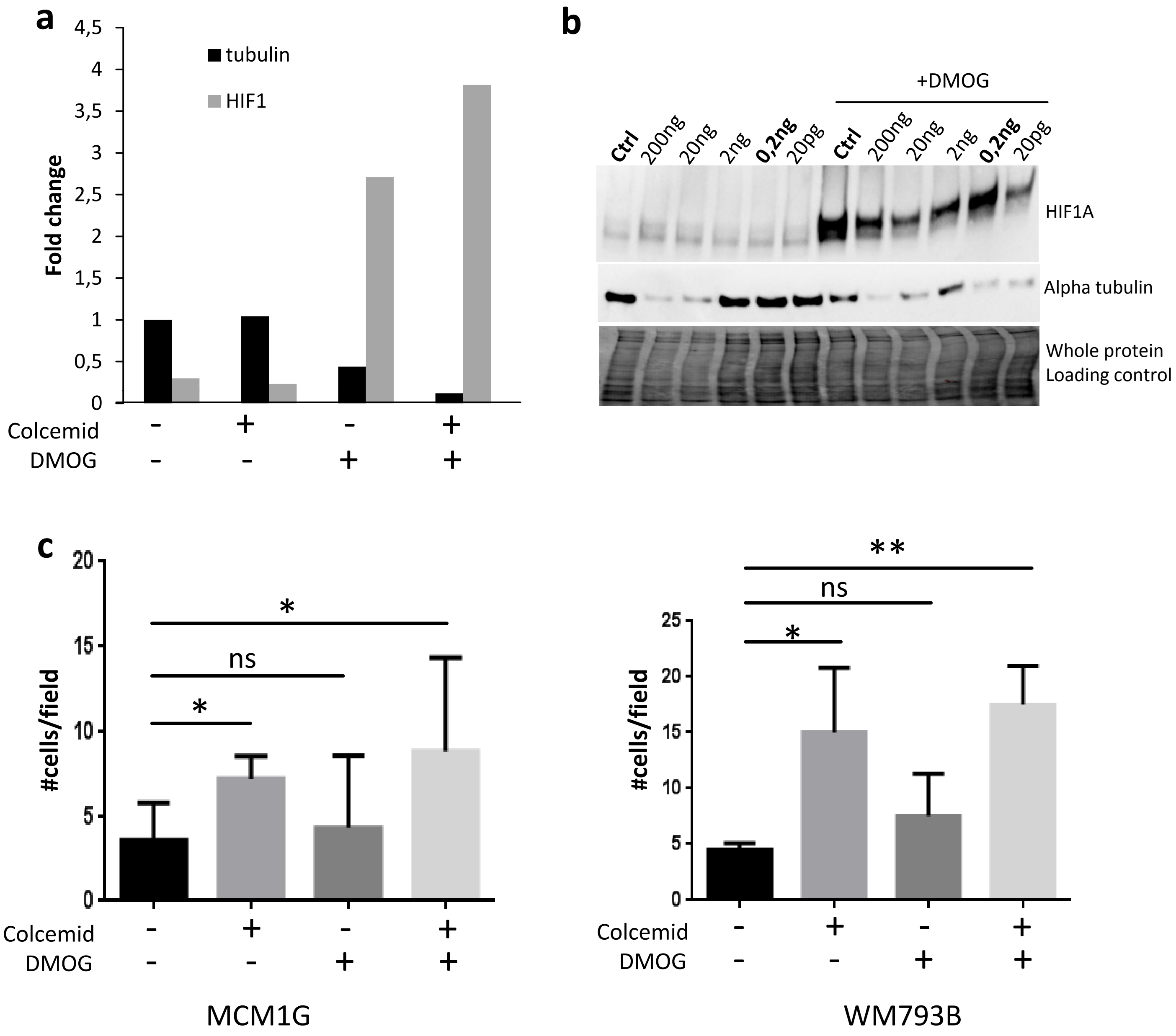

Legend to Supplementary Figure S2:

a) Normalized Western blot showing effects of colcemid with and without addition of DMOG on α-tubulin and HIF1A expression. b) Western blot image for HIF1a and tubulin and whole protein used as loading control. Melanoma samples were treated with decreasing colcemid concentrations with or without DMOG as indicated. Samples further used for ELISA in figure 5b are depicted in bold. c) Transwell migration of non-metastatic melanoma cells (left: MCM1G, right: WM793B) treated with colcemid and DMOG as indicated. Experiments were done in triplicates and 4 pictures/well were taken and counted. Number of cells per field are shown. p-values are shown in the graph as * p<0.05, ** p<0,01 and *** p<0,0001. ns=not significant.
